# The complete chloroplast genome of *Magnolia delavayi*, a threatened species endemic to Southwest China

**DOI:** 10.1080/23802359.2019.1660252

**Published:** 2020-07-16

**Authors:** Xiaodan Xu, Sensen Wu, Jingjing Xia, Bo Yan

**Affiliations:** Department of Environmental Art, Faculty of Art and Communication, Kunming University of Science and Technology, Kunming, China

**Keywords:** *Magnolia delavayi*, chloroplast genome, Illumina sequencing, phylogenetic analysis

## Abstract

*Magnolia delavayi*, a threatened plant endemic to Southwest China, is of great importance for landscaping because of its lotus-like creamy flowers. In this study, the complete chloroplast (cp) genome of *M. delavayi* was assembled based on the Illumina sequences. The cp genome of *M. delavayi* was 159,470 bp in length and contained a pair of inverted regions (IR, 26,409 bp) which were separated by the small single copy (SSC, 18,760 bp) and the large single copy (LSC, 87,892 bp) regions. It encoded 132 genes including 86 protein-coding genes, 37 tRNA genes, and eight rRNA ribosomal genes. The overall AT content of *M. delavayi* cp genome is 60.7%. The maximum likelihood phylogenetic analysis revealed that the species of *M. delavayi* was isolated first among the genus *Magnolia*. This result will be helpful for the conservation and phylogeny programs of the genus *Magnolia*.

*Magnolia delavayi* Franch., an evergreen tree in family Magnoliaceae, is endemic to Southwest China (Li et al. 2017). The species is of great importance for landscaping as well as medicine (Cao et al. 2004). It has been cultivated in Buddhist temples in Southwest China for hundred years because its attractive lotus-like creamy flowers (Figure 1) are regarded as the flowers of ‘Udumbara’ in Buddhist culture (Lin et al. 2003). In recent decades, more and more plants of *M. delavayi* has been used for local urban greening (Lin et al. 2003). Although it ban be propagated by grafting and tissue culture (Tang et al. 2014), the wild resources still go through excessive anthropogenic destruction by transplanting. Furthermore, the *M. delavayi* has extremely low seed-setting rate due to the special characteristics of blooming and pollination (Gong and Wu 1998; Li et al. 2017). At present, the species of *M. delavayi* has been seriously declined, and has been classified as “Least Concern” in the IUCN Red List of Threatened Species (Rivers and Wheeler 2014). Therefore, the *M. delavayi* should be protected effectively to avoid endangered even extinction. Previous studies of *M. delavayi* mainly focused on its basic biology, such as ovule number (Gong et al. 1999), and pollen germination (Li et al. 2017), but no complete chloroplast (cp) genome of *M. delavayi* has been reported. Here, we assembled the cp genome of *M. delavayi* as its basic conservation genetic resources.

**Figure 1.**
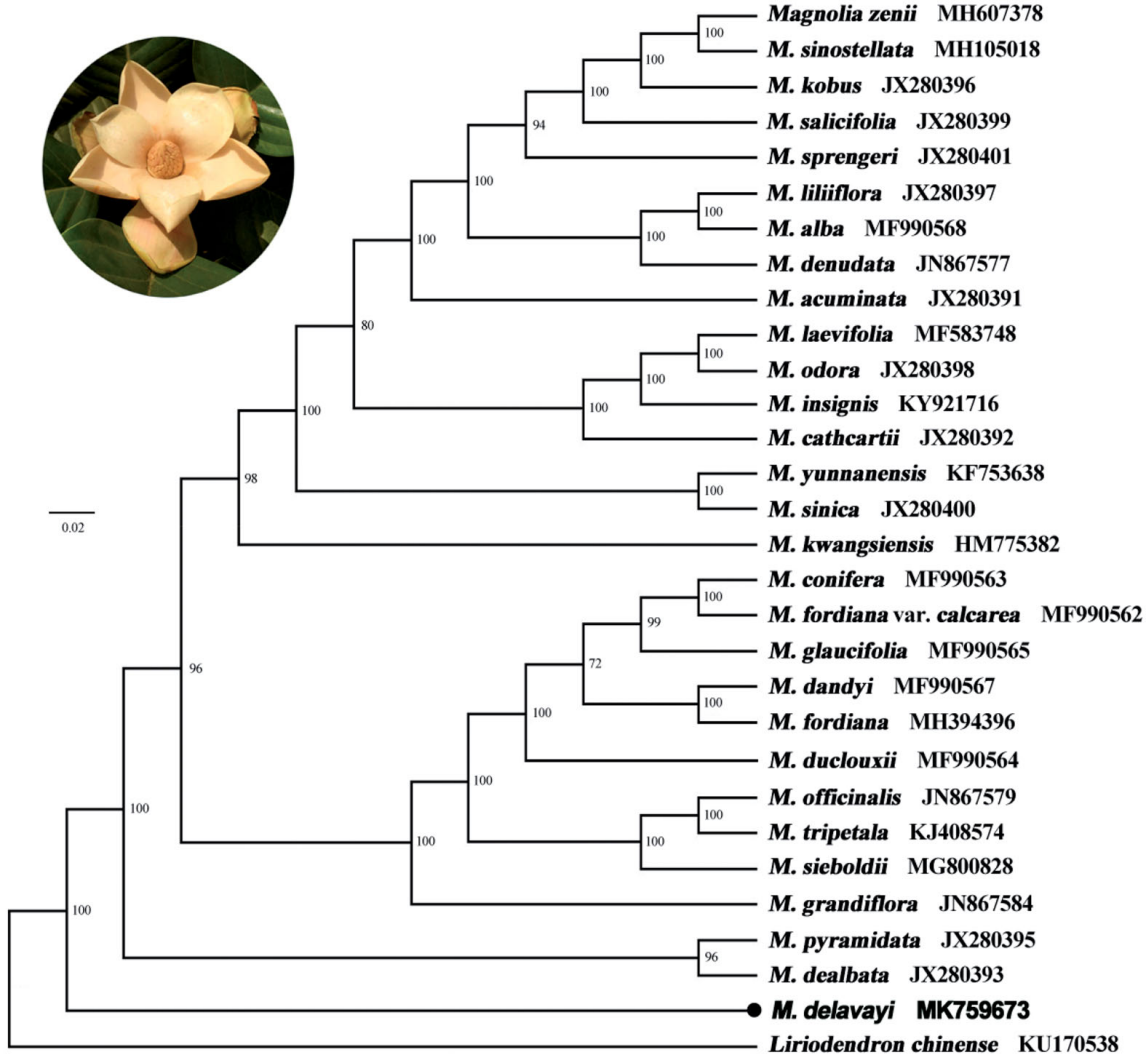
Maximum-likelihood phylogenetic tree for *Magnolia delavayi* based on 30 complete chloroplast genomes. The number on each node indicates bootstrap support value.

The total DNA from leaf tissue samples of a single individual (Location: N25°23′52″, E102°40′18″. Specimen voucher: Xu2018099, stored in the Herbarium of Kunming University of Science and Technology) was extracted with a modified CTAB method (Doyle and Doyle 1987). Illumina libraries were constructed, and high-throughput sequencing was carried out on the Illumina HiSeq X Ten sequencing system following the manufacturer’s protocol (Illumina, CA, USA). Approximately 2.0 Gb of clean reads data were generated after trimming with Trimmomatic v0.36 (http://www.sadellab.org/cms/index.php?page=trimmomatic) (Bolger et al. 2014). A combination of de novo assembly and reference-assisted mapping was applied to assemble the cp genome using Geneious R10 software (Biomatters Ltd., Auckland, New Zealand). Finally, the annotated cp genome sequence was submitted to GenBank (accession number MK759673).

The cp genome of *M. delavayi* was 159,470 bp in length and contained a pair of IR regions (26,409 bp) which were separated by a SSC region (18,760 bp) and a LSC region (87,892 bp). Whole cp genome encoded 132 genes including 86 protein-coding genes, 37 tRNA genes, eight rRNA ribosomal genes. In these genes, eight genes (*ndh*A, *pet*B, *rpo*C1, *rps*16, *trn*C-ACA, *trn*G-UCC, *trn*K-UUU, and *trn*L-UAA) harbored one intron and six genes (*clp*P, *ndh*B, *rpl*2, *trn*A-UGC, *trn*G-UUC, and *ycf*3) had two introns. Most of genes occurred in a single copy, while six PCGs (*ndh*B, *rpl*2, *rpl*23, *rps*7, *ycf*2, and *ycf*15), seven tRNA genes (*trn*A-UGC, *trn*I-CAU, *trn*I-GAU, *trn*L-CAA, *trn*N-GUU, *trn*R-ACG, and *trn*V-GAC), and four rRNA genes (*rrn*4.5, *rrn*5, *rrn*16, and *rrn*23) in IR regions were duplicated. The overall AT content of *M. delavayi* cp genome was 60.7% and the corresponding values in LSC, SSC, and IR regions were 62.0, 65.6, and 56.8%, respectively.

A total of 29 cp genomes of Magnoliaceae together with the obtained cp genome in this study were utilized to clarify the phylogenetic position of *M. delavayi*, by the outgroup of *Liriodendron chinense*. All of the cp genome sequences were aligned using MAFFT (Katoh and Standley 2013) by the software Geneious R10. A maximum likelihood analysis was performed with the RAxML software using 1000 bootstrap replicates. The phylogenetic tree revealed that the species of *M. delavayi* was isolated first among the genus *Magnolia* with the support rate of 100% (Figure 1). This phylogenetic result enhanced the study by Shen et al. (2018) using complete cp genomes but was not consistent with the analysis by Kim et al. (2001) using cp *ndh*F and Nie et al. (2008) by nuclear genes.

The first report of complete cp genome in *M. delavayi* will be a valuable resource for the future studies in conservation genetics, phylogeny, and breeding in *Magnolia.*
